# Tricolor R/G/B Laser Diode Based Eye-Safe White Lighting Communication Beyond 8 Gbit/s

**DOI:** 10.1038/s41598-017-00052-8

**Published:** 2017-01-31

**Authors:** Tsai-Chen Wu, Yu-Chieh Chi, Huai-Yung Wang, Cheng-Ting Tsai, Yu-Fang Huang, Gong-Ru Lin

**Affiliations:** Graduate Institute of Photonics and Optoelectronics, and Department of Electrical Engineering, National Taiwan University, Taipei, 10617 Taiwan

## Abstract

White light generation by mixing red, green, and blue laser diodes (RGB LDs) was demonstrated with Commission International de l’Eclairage coordinates of (0.2928, 0.2981), a correlated color temperature of 8382 K, and a color rendering index of 54.4 to provide a maximal illuminance of 7540 lux. All the white lights generated using RGB LDs were set within the risk group-1 criterion to avoid the blue-light hazard to human eyes. In addition, the RGB-LD mixed white light was diffused using a frosted glass to avoid optical aberration and to improve the performance of the lighting source. In addition, visible light communication (VLC) by using RGB-LD mixed white-light carriers and a point-to-point scheme over 1 m was performed in the directly modulated 16-QAM OFDM data format. In back-to-back transmission, the maximal allowable data rate at 10.8, 10.4, and 8 Gbps was determined for R, G, and B LDs, respectively. Moreover, the RGB-LD mixed white light-based indoor wavelength-division multiplexing (WDM)-VLC system yielded a total allowable transmission data rate of 8.8 Gbps over 0.5 m in free space. Such a high-speed RGB-LD mixed WDM-VLC system without any channel interference can be used to simultaneously provide data transmission and white lighting in an indoor environment.

## Introduction

The coverage of indoor lighting and visible light communication (VLC) systems is one of the promising approaches for developing smart homes because it can simultaneously provide compact lighting fidelity and convenient data transmission functionality^1–6^. Compared with phosphor-coated blue LEDs, the use of red, green, and blue (RGB) light-emitting diodes (LEDs) to generate a white-light source is an attractive lighting system with a high color rendering index (CRI)^7–9^. Such a high-directional white-light source generated using RGB LEDs can be used as desk lamps or daylight lamps. In 2002, Muthu *et al.* proposed the generation of a white LED source by mixing RGB LEDs of different luminances^10^. In 2008, Gilewski *et al.* reported the use of an optical feedback circuit to further control the light CRI and the luminance of the RGB LED mixed white-light source^11^. In 2010, Wang *et al.* reported the use of a novel control system to flexibly change the light color and luminous intensity of RGB-LED rendered white light^12^. In 2015, Hung *et al.* preliminarily constructed an RGB LED-based white light with multicolor rendered output by rotating the controllable mechanism^13^. To meet the data transmission demand, RGB LEDs within the white-lighting module were used to construct a wavelength-division multiplexing (WDM) VLC link. In 2012, Cossu *et al.* first demonstrated both 1.5-Gbps single-channel and 3.4-Gbps tricolor WDM transmissions through a 10-cm free-space channel by using an RGB-LED white-lighting module^14^. In 2013, Wang *et al.* presented an RGB-LED white-lighting source based WDM-VLC system with a total transmission data rate of 575 Mbps over 66 cm by employing the quadrature amplitude modulation (QAM)-orthogonal frequency division multiplexing (OFDM) with high spectral usage efficiency^15^. In 2014, Brandl *et al.* reported the use of a 680-nm vertical-cavity surface-emitting laser and a PIN photodiode (PD) to transmit a pseudorandom binary sequence at 3 Gbps over 19 m in free space^16^. By employing a high-sensitivity complementary metal-oxide-semiconductor (CMOS) avalanche PD (originally employed for single-photon detection), Kosman *et al.* successfully demonstrated an RGB LED-based VLC system in an ambient light of 1000 lux at 60 Mbps over 2 m in free space^17^. Recently, Wu *et al.* adapted the carrier-less amplitude and phase and OFDM data streams to directly encode the RGB LEDs for implementing a WDM-VLC system over 25 cm at maximal data rates of 3.2 and 2.9 Gbps, respectively^18^. Li *et al.* demonstrated the longest transmission distance of more than 70 cm by using an RGB-LED whit-lighting module-based WDM OFDM VLC system at 750 Mbps^19^. In 2015, Chen *et al.* proposed a hierarchical scheme to detect data transmission from rotatable RGB-LED arrays, which were developed for use as flash lamps for mobile-phone cameras^20^. In similar study, an optical camera communication based on the RGB-LED white-lighting flash lamp at a data rate of 150 bps was constructed^21^.

However, because of such shortcomings as narrow modulation bandwidth and low-coherent and nondirectional spontaneous emission, the LED-based VLC system can rarely perform high-speed transmissions with a large capacity and over a long distance. Therefore, laser diodes (LDs) of different colors with a high modulation bandwidth and high output power are considered one of the optimal solutions for constructing a high-speed and long-range VLC system^22–26^. In 2007, Hu *et al.* demonstrated a 10-Mbps VLC link with a transmission distance of up to 300 m by using a 650-nm LD^27^. The construction of a 500-Mbps WDM-VLC system over 10 m by using red and green laser pointers was reported in 2012^28^. Furthermore, in 2015, Chi *et al.* reported a 450-nm gallium nitride-LD based VLC for a 9-Gbps QAM-OFDM transmission over 5 m in free space^29^. To combine VLC with indoor white-lighting, the tricolor RGB LDs were employed to generate white light after divergence by using a diffuser, which can be applied to both indoor lighting and communication fidelities. In 2011, Neumann *et al.* reported on the mixing of red, green, blue, and yellow light components generated from LDs to obtain a highly bright white light^30^. In 2013, Soltic *et al.* proposed the optimization of an RGB LD-based white-lighting system by using the delta-function spectra^31^. In 2015, Haas *et al.* proposed that by using 36 parallel data streams, a transmission data rate of up to 100 Gbps can be achieved in a RGB LD-based WDM-VLC system^32^. According to these studies, implementing versatile RGB LD-based WDM-VLC links, such RGB LD-based VLC modules, has great potential for providing sufficiently bright white light for indoor lighting, which concurrently exhibits the capability to transmit data in a very short duration.

In this study, a tricolor set of RGB LDs was established to construct an indoor white-lighting WDM-VLC system with an allowable transmission raw data rate of 8.8 Gbps in total, and a maximal transmission distance of 0.5 m with forward-error correction (FEC) qualifications was demonstrated. The white light was generated by combining the RGB LD outputs with three dichroic mirrors and diverging them by using a frosted glass. The illuminance, the Commission International de l’Eclairage (CIE) coordinates, and the correlated color temperature (CCT) of the tricolor RGB LD-generated white light were measured. To avoid the risk of human-eye damage caused by the residual blue light component within the RGB-LD output, the power of the residual blue light was further detected using a spectrally filtered power meter to assess whether it passed the risk-group hazard to human eyes as defined in the standard IEC 62471 specifications. Before evaluating the performance of the tricolor RGB LD-based indoor-lighting WDM-VLC, each R, G, and B LD was individually employed to analyze the point-to-point VLC over 1 m to demonstrate the ultimate transmission performance of a single LD. The biased currents of the RGB LDs were optimized to upgrade their transmission performances for directly modulated 16-QAM OFDM data. The average error vector magnitude (EVM), signal-to-noise ratio (SNR), and bit-error-rate (BER) of the received 16-QAM OFDM data with maximal allowable bandwidth carried by RGB LDs were determined. In addition, the maximal transmission data rate delivered by the directly encoded RGB-LD set was characterized. Moreover, the variation in maximal allowable transmission capacity of the blue LD with changing CCT of the RGB-LD generated white light was also comprehensively analyzed.

## Results

### Characteristics of RGB LDs and the delivered white-light source

**Figure 1:**
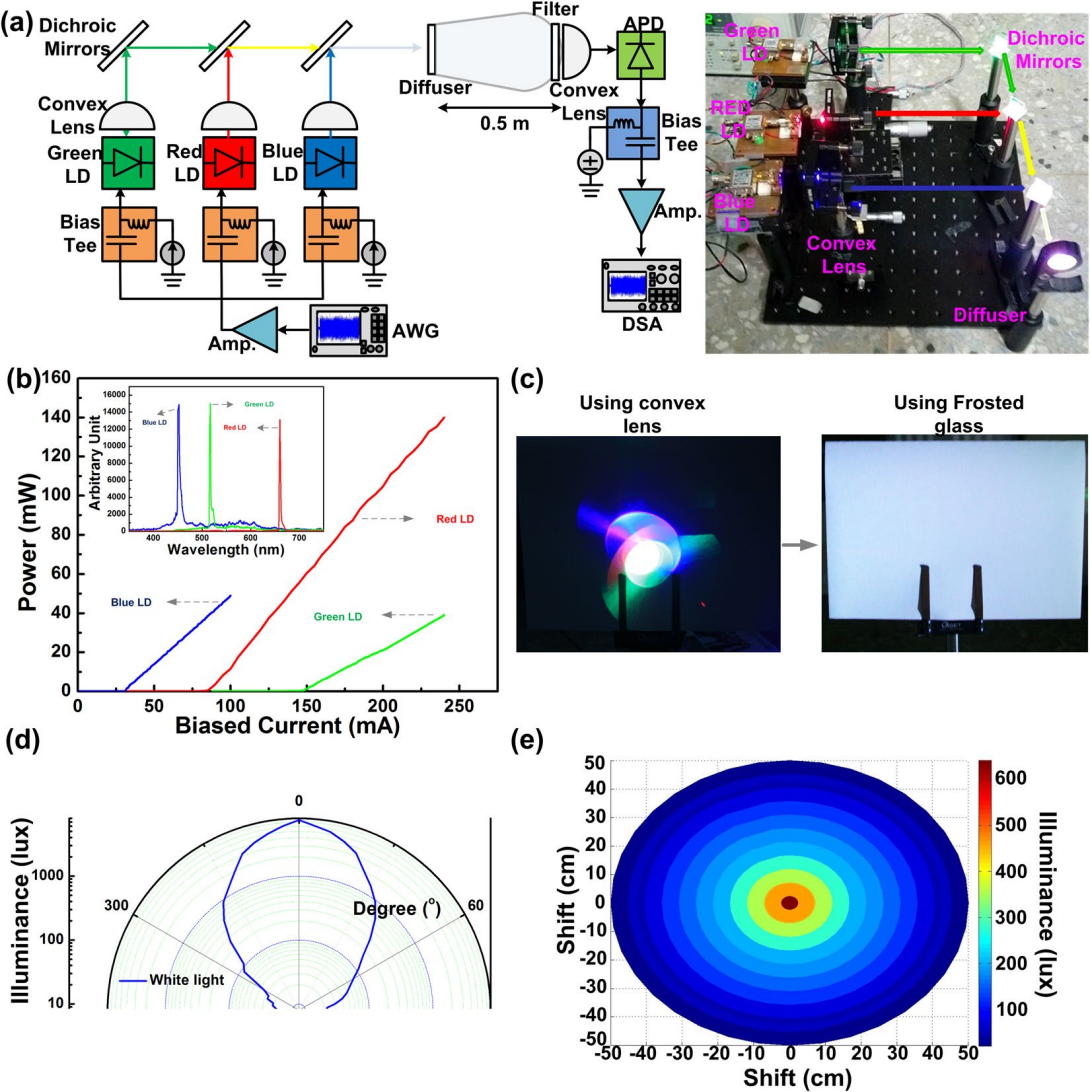
RGB-LD mixed white light source. (**a**) Experimental arrangement of the RGB LD-based indoor-lighting WDM-VLC system. (**b**) P-I response and optical spectra of RGB LDs. (**c**) Images of RGB-LD mixed white light with various diffusers. (**d**) Illuminance of the generated white light at various received angles. (**e**) Illuminance of white light at various received areas.

The experimental arrangement of mixed RGB LDs for both indoor luminescent lighting and data transmission is illustrated in Fig. 1(a). The power-to-current (P-I) response and related optical spectra of RGB LDs are presented in Fig. 1(b). The RGB LDs exhibited threshold currents of 85/148/31 mA and *dP*/*dI* slopes of 0.9, 0.4, and 0.7 W/A, with corresponding external quantum efficiencies of 0.32, 0.14, and 0.25, respectively. After lasing, their peak wavelengths were at 659, 516 and, 452 nm, with related full-width at half-maximum of 5.43, 3.26, and 6.52 nm, respectively. For emanative white-lighting after tricolor laser light mixing, the collimated RGB-LD beam was simply diverged using a convex lens at the first stage, as shown in the left panel of Fig. 1(c). Notably, such thick lens-induced beam divergence induced severe optical aberration, and the convex lens failed to completely mix and uniformly diffuse the collimated mixed RGB laser beam. Therefore, a frosted glass plate with a double-sided roughened surface was employed to replace the convex lens as the divergent diffuser, which perfectly mixed and diffused the collimated RGB laser beam to deliver a pure white light, as shown in the right panel of Fig. 1(c). To examine the angle-dependent illuminance, the RGB-LD mixed and diffused white-light source was mounted at the center of a rotating stage, and its illuminance was measured 10 cm from the source point by whirling the beam orientation (Fig. 1(d)). Consequently, a maximal illuminance of 7540 lux was observed at an orientation angle of 0°. To meet the required illuminance of 600 lux in a reading environment, the RGB-LD mixed white light exhibited a diverged angle of ±35°. For the general purpose of indoor lighting, the RGB-LD mixed/diffused white-light source can also provide an illuminance of 100 lux within ±55° diverged angle, as required for indoor lighting. By increasing the measured distance between the RGB-LD mixed/diffused white-light source and illuminometer to 50 cm, the illuminance of the lighting area was analyzed by shifting the position of the illuminometer away from the collimated axis (Fig. 1(e)). Notably, the illumination lux of the center degraded to half at an area of 407 cm^2^. At an irradiated area of 1.26 × 1.26 cm^2^, the received illuminance can be larger than 600 lux for reading. For indoor lighting, the irradiated area can be increased to 36 × 36 cm^2^. For general lighting purposes, the illuminance of such an RGB-LD mixed white-light source can be further improved by increasing the bias current of individual R, G, and B LDs; however, a trade-off on degraded communication performance is also raised at enlarging bias of the R, G, and B LDs.

**Figure 2:**
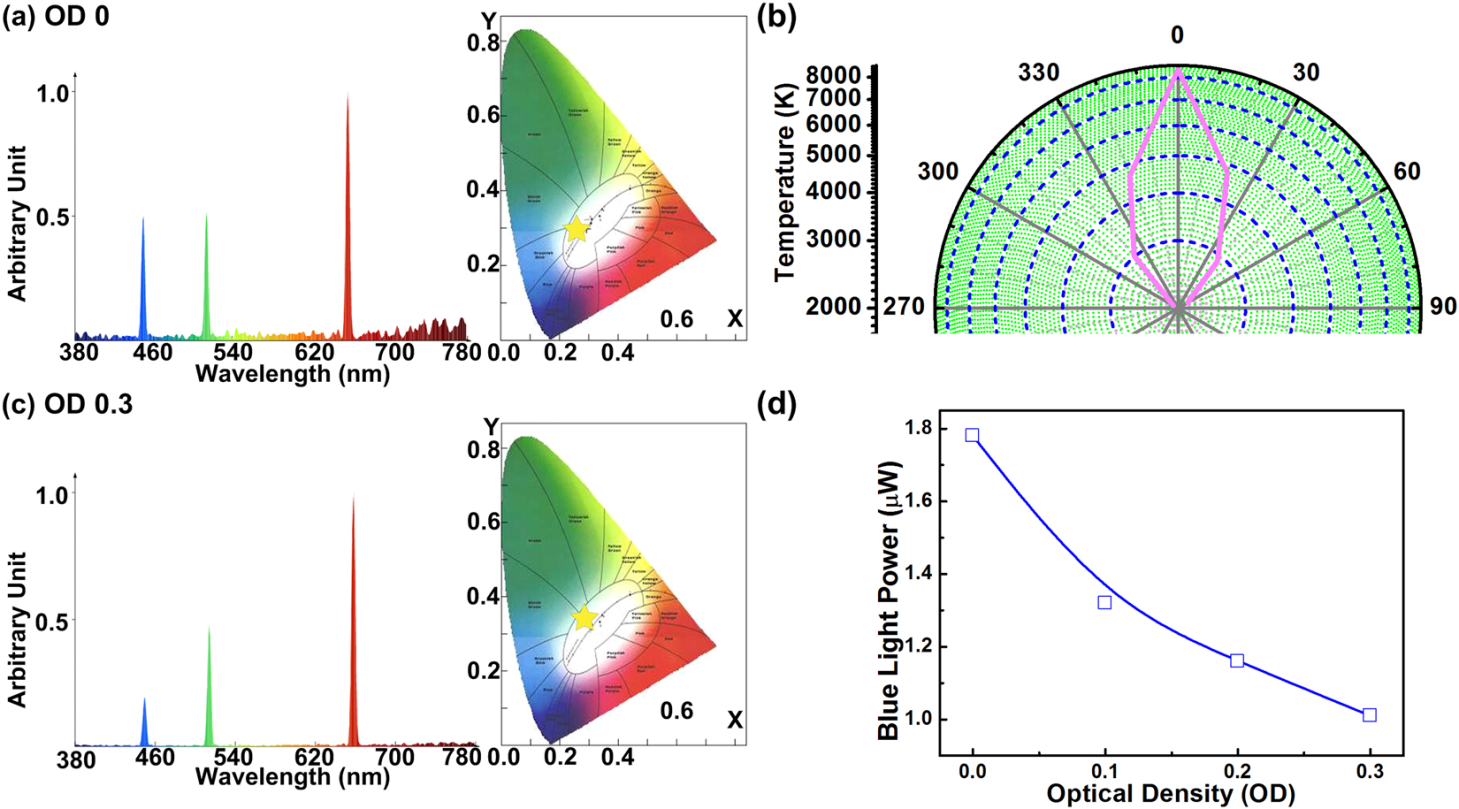
Lighting performance of RGB-LD mixed white-light source. (**a**) Optical spectra and CIE1931 chromaticity plots of the RGB-LD mixed white-light source without an OD filter. (**b**) CCT uniformity of the RGB-LD mixed white light source at various received angles. (**c**) Optical spectra and CIE1931 chromaticity plots of the RGB-LD mixed white-light source with an OD filter. (**d**) Power of blue laser beam within RGB-LD mixed white light at different OD values.

The optical spectra, CIE coordinates, and CCT values of RGB-LD mixed white light with and without the addition of the optical density (OD) filter are compared in Fig. 2. Without the OD filter use, the original RGB-LD mixed white light exhibited CIE coordinates, CCT value, and CRI of (0.2928, 0.2981), 8382 K, and 54.4, respectively (Fig. 2(a)). The CCT uniformity of the RGB-LD mixed white light source in the illumination area is presented in Fig. 2(b). The highest CCT of 8382 K is obtained at orientation angle of 0°. When increasing the diverged angle to ±15°, the CCT is decreased from 8382 K to 5500 K. The CCT keeps decreasing down to 2000 K with further when enlarging the divergent angle to ±60°. Certainly, the appropriate selection of RGB wavelengths would determine the CRI with compromised luminous efficiency. In general, the red wavelength is set at 625–630 nm for LED applications. Selecting a red LD at a longer wavelength improves the color rendering reduces the luminous efficiency owing to the degrading sensitivity of the human eye. To slightly weaken the power of the blue laser beam to reduce the hazard of the proposed white light to the human eye, the 0.3-OD filter was fixed in front of the blue LD, which altered the CIE coordinates, CCT value, and CRI to (0.2938, 0.3513), 7275 K, and 22.2, respectively (Fig. 2(c)). Attenuating the blue-light power instead of decreasing the bias current ensured that a sufficient modulation bandwidth was obtained for carrying the OFDM data in this study. According to IEC 62471, the hazard induced by blue light on the human eye is a critical concern; therefore, the residual power of the blue light should be confined to less than that defined by the risk group-1 (RG1) criterion for white light in a general lighting situation^33^. According to the RG1 criterion, the exposure limit of the human eye to blue light is 100–10000 W/m^2^sr. When the human eye is exposed to a point light source with brightness of <0.01 cd/m^2^, the pupil diameter (*D*_*Pupil*_) of the eye enhance to approximately 7 mm. Therefore, to evaluate the hazard of the blue laser beam to the human eye, the pupil diameter of the eye was assumed to be 7 mm^34^. Considering a free-space distance (*d*) of 0.5 m, the irradiated angle from the proposed white-light source to the pupil of the human eye can be calculated using *α* = *2*tan^−1^(*D*_*pupil*_/*2d)*. By setting *d* = 0.5 m and *α* = 1.6° (0.028 rad), a related solid angle (Ω = *πα* ^2^ */*4) of 6.16 × 10^−4^ sr was obtained. Furthermore, the power of the blue light component within the proposed white-light source was 1.78 μW, which corresponds to a related irradiance (*I*) of 62.95 × 10^−3^ W/m^2^, considering that the entire pupil of the human eye was exposed to the whole irradiation. Subsequently, the radiance (*L*_*s*_) of the RGB-LD mixed white-light source can be obtained as *L*_*s*_ = Ω/*I* = 62.95 × 10^−3^/6.16 × 10^−4^ = 102.19 W/m^2^ sr, which satisfies the IEC 62471 RG1 criterion. The residual power of the blue laser beam within the RGB-LD mixed white light when using a 0.3-OD filter is presented in Fig. 2(d). By attenuating the power and intensity of the blue light component to 1.01 μW and 35.72 × 10^−3^ W/m^2^ with the use of a 0.3-OD attenuator, respectively, the corresponding *L*_*s*_ can be reduced to 57.98 W/m^2^sr, which meets the RG0 (0–100 W/m^2^sr) criterion.

### Individual R, G, and B LD-based point-to-point VLC system

**Figure 3:**
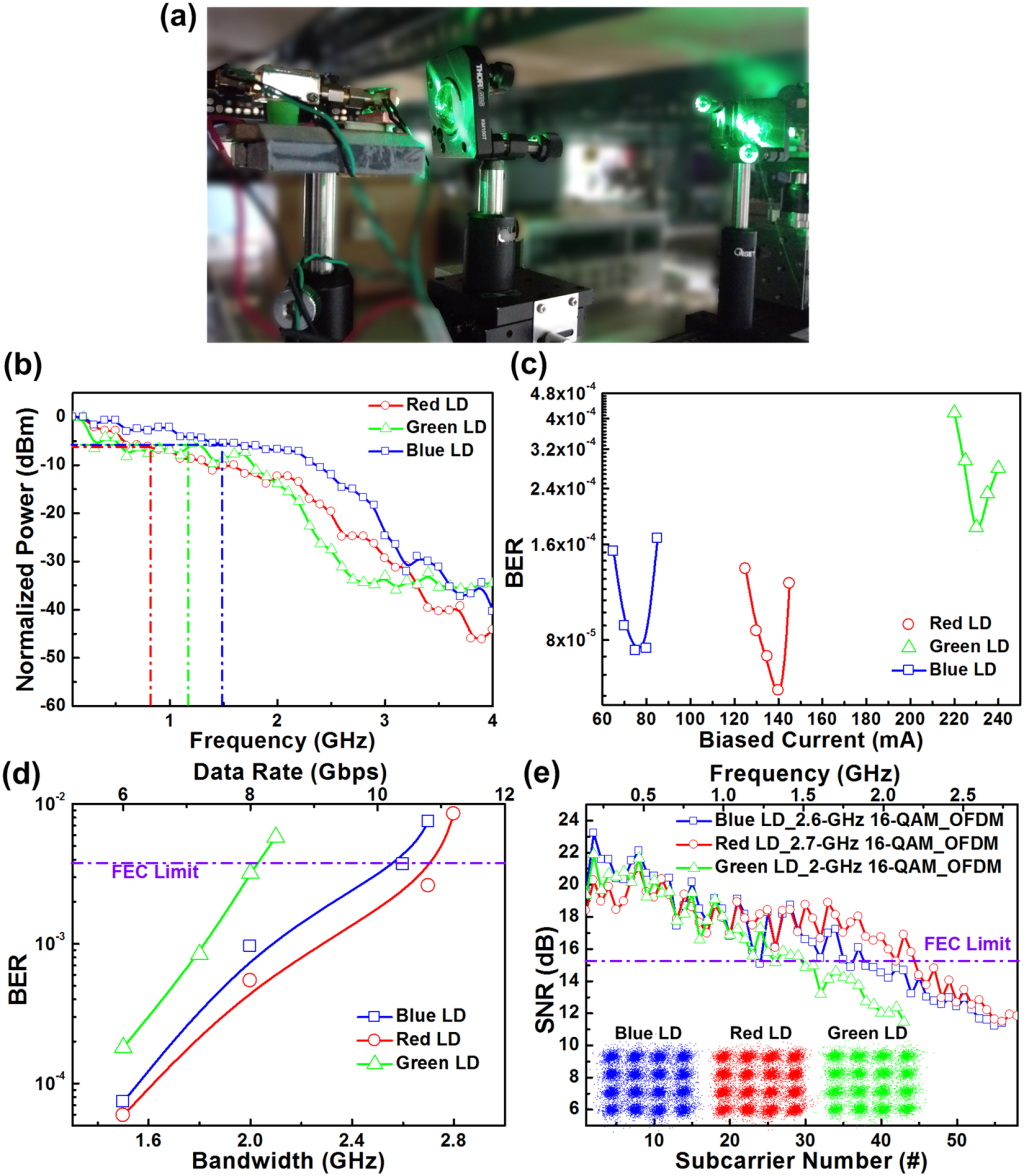
Individual R, G, and B LD-based point-to-point VLC system. (**a**) Illustration of green LD-based point-to-point VLC system. (**b**) Frequency responses of RGB LDs. (**c**) Received BERs of 6-Gbps 16-QAM-OFDM data transmitted by RGB LDs at various bias currents. (**d**) Average BERs of 16-QAM-OFDM data at various OFDM bandwidths transmitted by the individual R, G, and B LDs. (**e**) The subcarrier SNR response and corresponding constellation plots of individual R, G, and B LDs transmitted 16-QAM-OFDM data after a 1-m free-space transmission.

To verify the transmission capacity of individual R, G, and B LDs, a 1-m long point-to-point VLC system was established (Fig. 3(a)). The normalized frequency responses of individual R, G, and B LD transmitter at bias currents of 135 (1.6I_th_), 230 (1.6I_th_), and 75 (2.4I_th_) mA, respectively, are presented in Fig. 3(b). Under a direct modulation with a small signal with a power of 0 dBm, the RGB LDs transmitted sinusoidal-wave signals after the 0.5-m free-space transmission was received by the same PIN PD with a 3-dB bandwidth of 3 GHz and analyzed by the same RF spectrum analyzer (Agilent, 85651). Therefore, the PIN PD exhibited different responsivities at different wavelengths; the red LD exhibited the highest throughput intensity among three individual LD transmitters before normalization. In addition, the blue LD revealed a more favourable flatness at all frequency ranges than did the red and green LDs. Notably, the 6-dB modulation bandwidths of the individual R, G, and B LDs were determined as 0.8, 1.2, and 1.5 GHz, respectively. For DC bias optimization, a 6-Gbps 16-QAM OFDM data with a bandwidth of 1.5 GHz was used for directly modulating the RGB LDs (Fig. 3(c)). During the experiment, EVM, which indicates the effects of intensity and phase noises on the transmitted OFDM data, was used to obtain the SNR and BER for evaluating the transmission performances of proposed white-lighting communication system. In addition, the subcarrier SNRs response can assist in analyzing and optimizing the combined frequency response of all the used components. Moreover, the BER is the most common parameter for evaluating the transmission performance of a communication system. To estimate the performance of the 16-QAM-OFDM data transmission by RGB-LD mixed white light, the required EVM of 17.3%, SNR of 15.2 dB and BER of 3.8 × 10^−3^ were set for meeting the FEC criterion. To avoid data clipping below the threshold condition, the bias current was increased from 125 to 140 mA for the red LD to reduce the received BER from 1.3 × 10^−4^ to 5.9 × 10^−5^. When the bias was further increased to 145 mA, BER was inversely degraded to 1.21 × 10^−4^ because of the reduced modulation depth after direct modulation of the high-level biased LD. Although the frequency responses of the blue and green LDs were more favourable than that of the red LD, the responsivity of the PIN PD at the red wavelength was markedly higher than that of the blue and green LDs; this results in similar BER trends for different colored LDs. At optimized bias currents of 230 and 75 mA for the green and blue LDs, respectively, the received BERs improved to 1.81 × 10^−4^ and 7.4 × 10^−5^, respectively. Notably, the BERs at all conditions already passed the FEC criterion required BER of 3.8 × 10^−3^ ^35–37^. Subsequently, the covering bandwidth of the used 16-QAM OFDM data was increased to determine the optimization of the transmission capacity of individual R, G, and B LDs in the 1-m point-to-point VLC system (Fig. 3(d)). Increasing the data bandwidth from 1.5 to 2.7 GHz reduced the average BER from 5.9 × 10^−5^ to 2.6 × 10^−3^ for the red LD transmitted QAM-OFDM data, which provided a FEC qualified data rate of only 10.8 Gbps. Notably, the obtained BER was degraded to 8.5 × 10^−3^ by continuously increasing the data bandwidth up to 2.8 GHz under the influence of limited bandwidth by LD and PIN PD. In comparison, the maximal allowable data bandwidths of green and blue LD carriers were 2 and 2.6 GHz with related BERs of 3.2 × 10^−3^ and 3.7 × 10^−3^, respectively. Consequently, the maximal transmission data rates of individual R, G, and B LDs were determined as 10.8, 8, and 10.4 Gbps, respectively. The SNR responses of the 16-QAM data at various OFDM subcarriers and corresponding constellation plots after transmission by individual R, G, and B LDs over 1 m in free-space are presented in Fig. 3(e). The red LD yielded a 10.8-Gbps data transmission with an average EVM of 16.6% and an average SNR of 15.6 dB. By contrast, the green LD supported a transmission at only 8 Gbps with a corresponding EVM and SNR of 17% and 15.4 dB, respectively, owing to its insufficient modulation bandwidth. Moreover, the blue LD yielded a comparable transmission at 10 Gbps with a corresponding average EVM of 17.4% and SNR of 15.2 dB. Notably, most commercially available R, G, and B LDs are intended for use in displays or DVDs (red) and not for high-frequency responses. Although the frequency response of RGB LDs may be upgraded by improving their device structure in the near future, currently, the manufacture of a cost-effective device with improved frequency response for practical application is different.

### RGB-LD mixed white light source-based 8.8-Gbps WDM-VLC system

**Figure 4:**
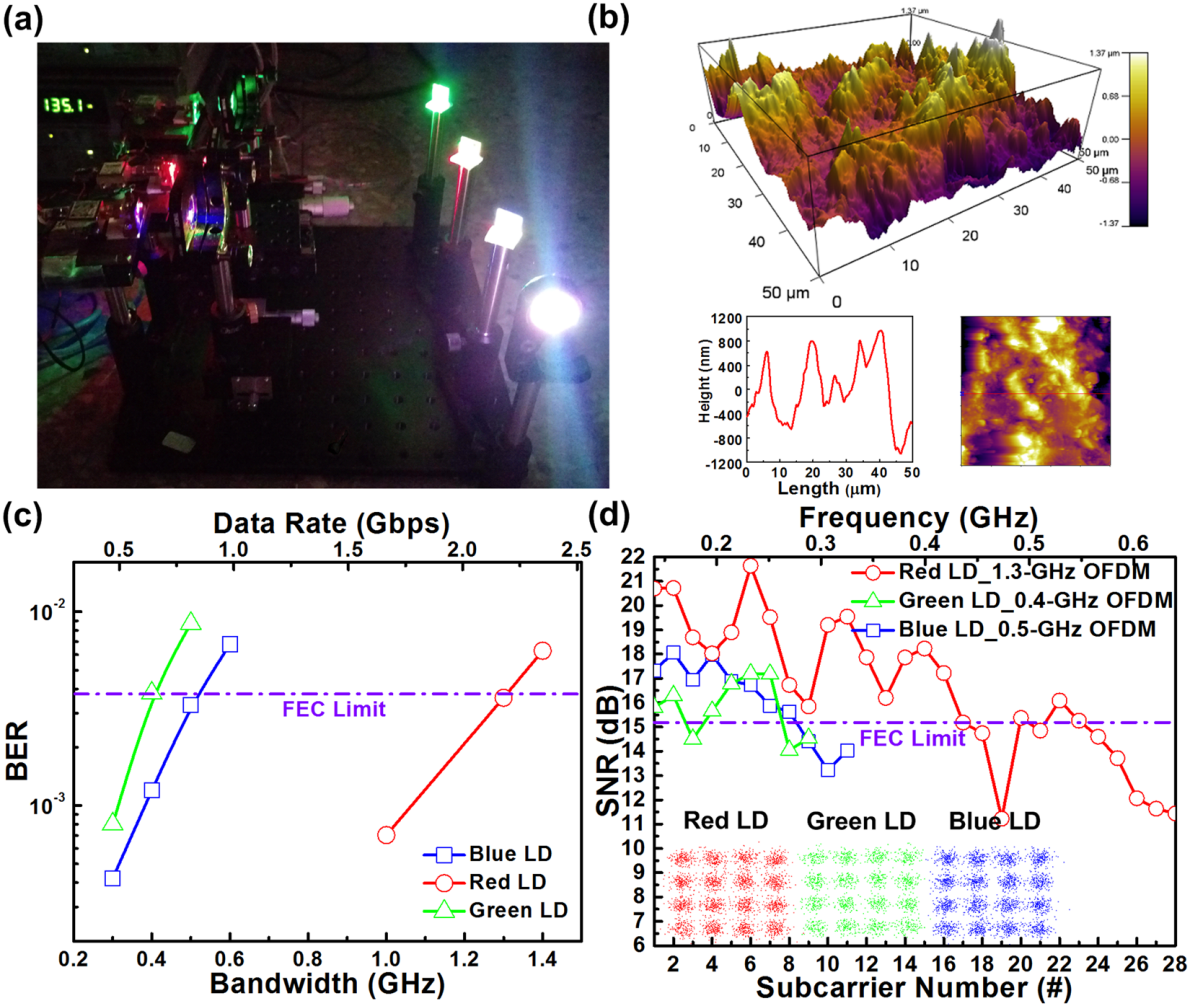
Mixed RGB LD-based WDM-VLC system. (**a**) Illustration of a mixed RGB LDs-based WDM-VLC system. (**b**) AFM image of the frosted glass. (**c**) BERs of 16-QAM-OFDM data transmitted by the red, green, and blue laser beams within the mixed white light. (**d**) The subcarrier SNR spectra and corresponding constellation plots of 0.5-m free-space transmitted 2-, 1.2-, and 2-Gbps 16-QAM-OFDM data carried by the RGB laser beams within the mixed white-light source.

To construct an RGB-LD mixed white-light carrier with WDM VLC for indoor white lighting, the laser beams of RGB LDs were collimated, and a frosted glass was employed to diffuse the mixed and collimated laser beam (Fig. 4(a)). Because the intensity of the RGB-LD mixed white-light source weakened after passing through the frosted glass, the PIN PD was replaced with an avalanche PD (APD, Hamamatsu, S9073) with a cut-off frequency of 0.9 GHz PD for improving the detectivity. Figure 4(b) presents the AFM characterization of the frosted glass with surface roughness of 492.3 nm (in root-mean square value). After free-space transmission over 0.5 m, the transmission performance of individual R, G, and B LDs was characterized using the free-space bandpass filter (Fig. 4(c)). To achieve the FEC-required BER of 3.8 × 10^−3^, the maximal allowable data bandwidths of R, G, and B LDs of 1.3, 0.4 and 0.5 GHz with associated transmission capacities of 5.2, 1.6, and 2 Gbps, respectively, were used. This provided a total raw data rate of up to 8.8 Gbps for the proposed RGB-LD mixed white-lighting WDM-VLC system. In addition, the SNR spectra and corresponding constellation plots of 0.5-m free-space transmitted 16-QAM-OFDM data delivered by the R, G, and B LD carriers after mixing into the white light are presented in Fig. 4(d). For the RGB-LD mixed white-light source, the red/green/blue laser beams can achieve 5.2-/1.6-/2-Gbps data rate with corresponding EVM, SNR and BER of 17.3%/17.4%/17.2%, 15.2/15.2/15.3 dB, and 3.6 × 10^−3^/3.8 × 10^−3^/3.5 × 10^−3^, respectively.

**Figure 5:**
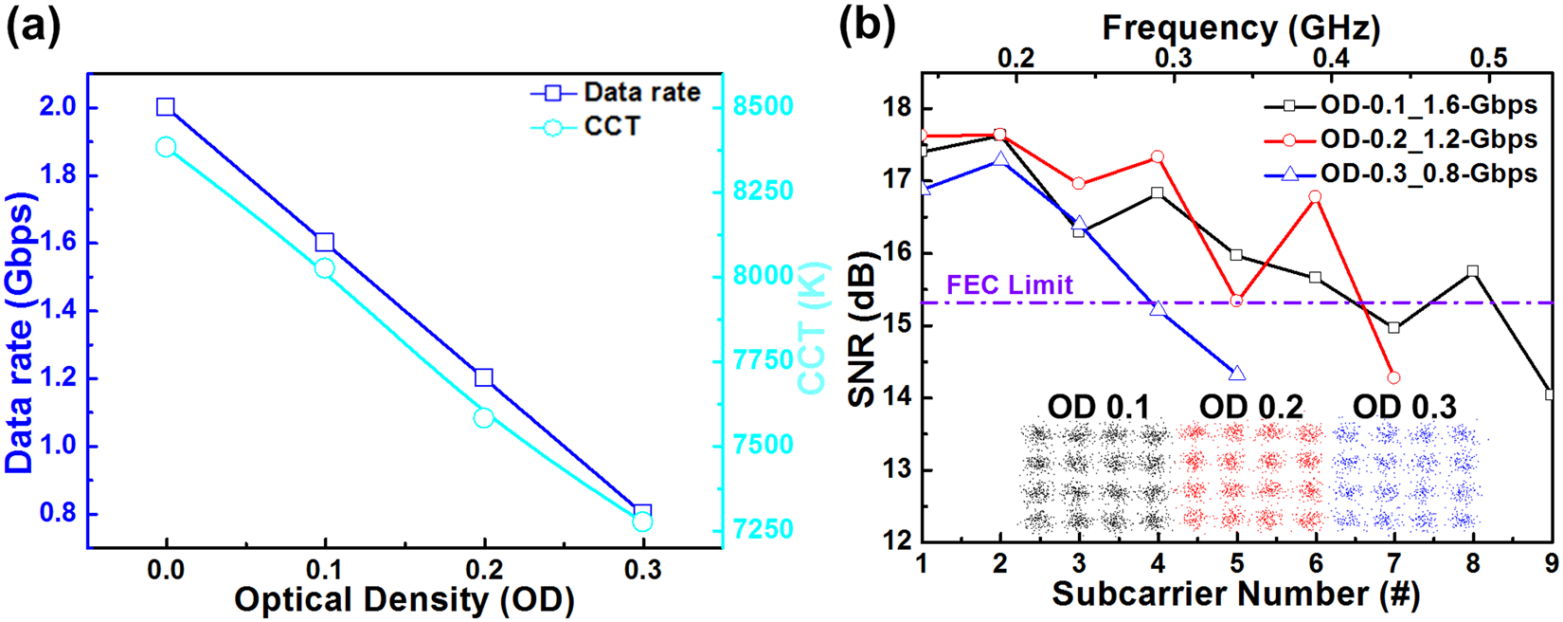
White-lighting and transmission performances of the blue laser beam within the mixed white light at various OD values. (**a**) Maximal allowable data rate of the blue LD and CCT of the white light at various OD values. (**b**) SNR spectra and corresponding constellation plots of 1.6-, 1.2-, and 0.8-Gbps data transmitted by the blue laser beam within the mixed white light at various OD values.

This study assessed the ultimate performance, such as the maximal allowable transmission capacity of the RGB-LD mixed white-light source, by employing a digital serial analyzer (DSA) with a high sampling rate (100 GS/s) for overcoming the frequency-selective fading of transmitted data. For practical application of the proposed RGB-LD mixed white-light source, the R, G, and B LDs facilitate the transmission of the 16-QAM-OFDM data with data rates of 5.2, 1.6, and 2 Gbps, respectively, which require sampling rates of only 5.2, 1.6, and 2 GS/s (four times the modulation bandwidth), respectively. These low sampling rates are relatively easy to be realized using cost-effective CMOS-integrated circuits or application-specific integrated circuits for mass applications. When adhering the OD filters with different OD values in front of the blue LD, the transmission capacity of the blue laser beam and the CCT value of the RGB-LD mixed white-light source consequently varied (Fig. 5(a)). When the OD value was increased from 0.1 to 0.3, the CCT value of the mixed white light decreased from 8024 to 7275 K, and the maximal allowable data rate delivered by the blue laser carrier inevitably decreased from 1.6 to 0.8 Gbps. Evidently, the SNR and constellation plots of blue LD-delivered 16-QAM-OFDM data also degraded with increasing OD values (Fig. 5(b)). Using the 0.1-OD filter enables a 1.6-Gbps QAM-OFDM transmission with an average EVM, SNR, and BER of 16.9%, 15.5 dB, and 3 × 10^−3^, respectively. Among all cases, the 0.3-OD filter provided the lowest data rate of only 0.8 Gbps, which provided an average EVM, SNR, and BER of 17%, 15.4 dB, and 3.3 × 10^−3^, respectively. The maximal transmission capacity of the RGB-LD mixed white light strongly correlated with the throughput power of the blue LD carrier. For warm white light, the blue light component weakened; however, the data transmission rate of the RGB-LD mixed white-lighting WDM-VLC system decreased.

## Discussion

The RGB-LD arrangement was employed to establish an indoor white-lighting communication system. The individual R, G, and B LD-based point-to-point VLC with corresponding transmission data rates of 10.8/10.4/8 Gbps was demonstrated with an allowable transmission distance over 1 m. Subsequently, light diffusion and beam divergence were demonstrated using a frosted glass plate. The 16-QAM-OFDM data transmitted at 10.8 Gbps by a single red LD exhibited an average EVM, SNR, and BER of 16.6%, 15.6 dB, and 2.6 × 10^−3^, respectively. In comparison, the individual blue and green LDs provided 10.4- and 8-Gbps 16-QAM-OFDM data transmissions with average EVMs of 17.4 and 17%, average SNRs of 15.2 and 15.4 dB, and BERs of 3.7 × 10^−3^ and 3.2 × 10^−3^, respectively. When a frosted glass plate was used, the diffused and diverged white light mixed by the tricolor RGB LDs exhibited a transmission raw data rate of 8.8-Gbps over 0.5 m in an indoor-lighting environment. The illuminance of the RGB-LD-generated white light measured at a distance of 10 cm was higher than the required 600 lux within ±35° for a reading lamp and higher than the required 100 lux within ±55° for an indoor-lighting bulb. According to the Chinese National Standard issue of CNS-12112-Z1044 in Taiwan, the indoor-lighting environment requires a luminance of <1000 lux. A general office room requires a luminance ranging from >200 to <700 lux. This proposed system exhibited a high performance and compatible specification in practical applications. By further increasing the measured distance up to 50 cm, the obtained illuminance of white light remained >600 lux within an irradiated area of 1.6 cm^2^ and >100 lux within the irradiated area of 1296 cm^2^. Moreover, the CIE coordinates and CCT of the tricolor RGB-LD-generated white light were (0.2928, 0.2981) and 8382 K, respectively, and the CCT of white light was further decreased to 7275 K by adhering an 0.3-OD filter on the blue LD output. Notably, all the white lights generated by RGB LDs at various biasing recipes were confined within the RG1 criterion limit. The red/green/blue LDs transmitted 16-QAM-OFDM data at a maximal allowable data rate of 5.2-/1.6-/2-Gbps Gbps with average EVMs of 17.3%/17.4%/17.2%, SNRs of 15.2/15.2/15.3 dB, and BERs of 3.6 × 10^−3^/3.8 × 10^−3^/3.5 × 10^−3^, respectively. Moreover, the 16-QAM-OFDM data transmission rate of the blue LD decreased from 1.6 to 0.8 Gbps when the CCT of the RGB-LD mixed white light decreased from 8024 K to 7275 K with an increase in the OD value of the OD filter from 0.1 to 0.3. The color temperature of the RGB mixed white-light source can be reduced by either attenuating the blue laser beam intensity or increasing the red and green laser beam intensities. However, such operations may reduce its maximal allowable transmission capacity because the bias currents of red, green and blue laser diodes have already been optimized to compromise the relative intensity noise suppression and the frequency response declination. In this study, a maximal allowable data rate of 8.8 Gbps was obtained for the proposed RGB mixed white-light source at a relatively high color temperature of 8382 K. When a 0.3-OD attenuator was adhered in front of the blue LD, the color temperature of the RGB mixed white-light source decreased to 7275 K, and its transmission capacity inevitably decreased to 4 Gbps. Although the CCT and blue light power of the RGB-LD-generated white light can be further reduced by adhering the OD filter on the blue LD to render the white light harmless to the human eye, the maximal allowable data rate of the 16-QAM-OFDM stream delivered by the blue LD is simultaneously compromised.

Table 1 presents a comparison of the transmission and lighting performance of the LD-based white-light sources proposed in previous studies. In 2015, Tsonev *et al.* used RGB LDs for white-lighting communication with individual LDs at a data rate of more than 4 Gbit/s^32^, which also achieved an illuminance of 843.69 lux for a free-space link over 0.3 m. In addition, Janjua *et al.* demonstrated an RGB-LD mixed white-light source^23^ to transmit 16-QAM-OFDM data as short as approximately 0.2 m at >4 Gbps with a CCT value of 5835 K; however, the diffuser used in the study was not discussed in detail. By combining a blue LD with a remote phosphor, Chun *et al.* demonstrated a white-lighting VLC system with a CCT value of 7092 K at 6.52 Gbps for a free-space link over 0.15 m^5^. Subsequently, Chi *et al.* proposed a white-lighting VLC with a total data rate of up to 5.2 Gbps over 0.6 m in free space at a CCT value of 5217 K by diverging the blue LD by using a phosphorous diffuser^24^. This is the simplest white-lighting VLC system; however, the high reflection/scattering/absorption of phosphor with long lifetime inevitably degrades the transmission performance. By using commercial R, G, and B LDs, the present study demonstrated the method of upgrading the total transmission capacity to 8.8 Gbps over 0.5 m and the maintaining blue light power slightly beyond the RG-0 criterion to avoid the hazard to the human eye. In addition, the appropriate roughness of the frosted glass was analyzed specifically, unlike in previous studies.

**Table 1:** Summary of lighting and transmission performance of LD-based white-light sources.

Group	Type of LD	Distance	Data Rate	CCT
Tsonev *et al.* ^32^	RGB	0.3 m	14 Gbps	—
Janjua *et al.* ^23^	RGB	0.2 m	4.4 Gbps	5835 K
Chun *et al.* ^38^	Blue + Phosphor	0.15	6.52 Gbps	7092 K
Chi *et al.* ^24^	Blue + Phosphor	0.6 m	5.2 Gbps	5217 K
This work	RGB	0.5 m	8.8 Gbps	8382 K

In contrast to other LED based white-lighting VLC approaches, the commercial R, G, and B-LD-based white-lighting VLC system provided more flexibility in CCT tunability. The R, G, and B LD with nearly monochromatic colored linewidth essentially supported the lighting with enhanced chromaticity after proportional mixing, which enables the reproduction of broadband colored lighting with its spanned chromaticity diagram broader than those of currently available sources. Because the original LD beam exhibited higher directionality with smaller beam divergence, which provides larger power density to risk from higher blue-light hazard than the LED beam. However, this drawback was overcome in our system by using a highly roughened frosted glass plate. Furthermore, the present study demonstrated an upgraded transmission capacity without the risk of the blue-light hazard to the human eye (close to the RG-0 criterion).

## Methods

### Experimental setup of the tricolor RGB LD-based WDM-VLC system

The experimental arrangement of the tricolor RGB LD**-**based WDM-VLC system comprised 450- (OSRAM, PL 450B), 520- (OSRAM, PLP 520), and 650-nm (Mitsubishi, LPC-836) LDs. Each LD was controlled at a room temperature of 25 °C by using a thermistor for temperature sensing and a thermoelectric cooler for heat dissipation. For the tricolor mixing for white-light generation, the outputs of RGB LDs were combined using three dichroic mirrors. First, the green laser light was reflected by the first dichroic mirror to combine with the red laser light reflected by the second dichroic mirror. Subsequently, yellow laser light was formed by the combined red + green output, which was further converted to a white light beam after mixing with the blue laser light reflected by the third dichroic mirror. Thereafter, the white laser light was diffused and diverged using a frosted glass to meet the requirement of divergent indoor lighting with a broadened emitting angle. The volume of the RGD-LD was 18,000 cm^3^ (length × width × height = 40 × 30 × 15 cm^3^). The illuminance of the white light was measured using an illuminometer (TECPEL, DLM-530). The CIE coordinates and CCT were also measured using an integrating-sphere-assisted spectrometer (OKTEK, GL-2). To construct the white-lighting WDM-VLC system, the electrical 16-QAM-OFDM data at a cyclic prefix ratio of 1/32 with a fast Fourier transform (FFT) size of 512 and different subcarrier numbers were exported from an arbitrary waveform generator (Tektronix 70001A) with 24-GS/s sampling rate. After passing through a broadband pre-amplifier (Picosecond, 5865A), the electrical 16-QAM-OFDM data was combined with the DC bias current through a bias tee (Mini-circuit, ZX85-12G-S+) to directly modulate the tricolor RGB LDs with identical data formats. After transmission through a 0.5-m free-space channel, the optical 16-QAM-OFDM transmitted data by individual R, G, and B LD was filtered out from the divergent white light by using a corresponding bandpass filter. Moreover, the delivered optical 16-QAM-OFDM data was focused into an avalanche PD (APD, Hamamatsu, S9073) with a diameter of 200 μm by using a convex lens, and then converted into electrical 16-QAM-OFDM data. Subsequently, the received electrical 16-QAM-OFDM data was amplified using a high-gain amplifier (Mini-circuit, ZKL-1R5+) and sampled using a 100-GS/s DSA (Tektronix, 71604C). Finally, the MATLAB program was used to decode and analyze the constellation plot, EVM, SNR, and BER of the received 16-QAM-OFDM data.

### Parameters used for evaluating the QAM-OFDM data transmission performance of the RGB-LD mixed white light

For OFDM decoding, the EVM was used as a quantitative value to evaluate the difference between the ideal and measured symbols of the transmitted QAM OFDM data, and it can be expressed as follows^39^:

1 $$EVM( \% )=\sqrt{\frac{1}{N{P}_{0}}\sum _{n=1}^{N}{|{S}_{r}(n)-{S}_{t}(n)|}^{2}}\times 100 \% $$

where *N* denotes the number of received symbols; *P*_*0*_ is the maximum normalized power of the ideal symbol; *S*_*r*_(*n*) is the normalized received *n* ^*th*^ symbol which is corrupted by Gaussian noise; *S*_*t*_(*n*) is the ideal transmitted value of the *n* ^*th*^ symbol. In principle, the SNR of the received OFDM data can be calculated from the average EVM by using the following equation^40^:

2 $$SNR\cong \frac{1}{EV{M}^{2}}$$

The subcarrier SNRs response can assist in analyzing and optimizing the combined frequency response of all the used components. Finally, the BER performance can be obtained using the following equation^41^:

3 $$BER\cong \frac{2(1-\frac{1}{\sqrt{M}})}{{\mathrm{log}}_{2}M}\times \{erfc[\sqrt{\frac{3SNR}{2(M-1)}}]+erfc[3\sqrt{\frac{3SNR}{2(M-1)}}]\}$$

where *M* denotes the QAM level. During the experiment, the EVM was used to obtain the SNR and BER for evaluating the transmission performance of the white-lighting communication system.
